# An Uncommon Presentation of Heyde’s Syndrome: Gastric Antral Vascular Ectasia in a Patient With Severe Aortic Stenosis

**DOI:** 10.7759/cureus.90506

**Published:** 2025-08-19

**Authors:** Calvin Jose, Issac Georgy, Thomas V Pulickal, Alan J Kannemkuzhiyil, Avaroth Krishnadas

**Affiliations:** 1 Internal Medicine, Good Shepherd Hospital, Wayanad, IND; 2 Internal Medicine, St. John's Medical College, Bangalore, IND; 3 Emergency Medicine, All India Institute of Medical Sciences, Bhubaneswar, Bhubaneswar, IND; 4 Family and Community Medicine, Good Shepherd Hospital, Wayanad, IND

**Keywords:** angiodysplasia, aortic stenosis, argon plasma coagulation, elderly anemia, endoscopy, gastric antral vascular ectasia, gastrointestinal bleeding, gave, heyde’s syndrome, von willebrand factor

## Abstract

Heyde’s syndrome signifies the link between aortic stenosis (AS) and bleeding due to angiodysplastic changes. While colonic angiodysplasia is commonly implicated, gastric antral vascular ectasia (GAVE) represents a rarer manifestation in this context. We report the case of a 72-year-old male with severe AS who had recurrent upper gastrointestinal bleeding due to GAVE. Endoscopic hemostasis was achieved with argon plasma coagulation (APC). This case illustrates the need for multidisciplinary intervention while managing such patients and makes a further case for recognizing GAVE in the context of Heyde’s syndrome.

## Introduction

Heyde’s syndrome highlights the combination of aortic stenosis (AS), acquired von Willebrand factor deficiency, and gastrointestinal bleeding (GIB) due to angiodysplasia. It was first described in 1958 by Edward C. Heyde [1]. The pathophysiology features shear-induced proteolysis of high-molecular-weight von Willebrand factor (vWF) multimers that leads to impaired platelet adhesion and results in bleeding diathesis [2,3]. Angiodysplasia is classically responsible, but gastric antral vascular ectasia (GAVE), or “watermelon stomach,” while rare, should be considered as a differential diagnosis for upper GIB in this situation [4,5]. Early recognition of GAVE in the context of Heyde’s syndrome is clinically important, as targeted endoscopic therapy and timely valve intervention can prevent recurrent bleeding and improve patient outcomes. Through this case report, we describe our experience with a patient presenting with gastrointestinal (GI) bleeding due to GAVE in the setting of AS, which is consistent with Heyde’s syndrome.

## Case presentation

A 72-year-old Indian man presented with two episodes of hematemesis over the preceding month, each characterized by the vomiting of approximately 200 mL of fresh blood, without syncope but associated with melena. He had a history of AS, moderate aortic regurgitation (AR), hypertension, and benign prostatic hyperplasia. He denied long-term use of nonsteroidal anti-inflammatory drugs or anticoagulants. There was no history of alcohol use, liver disease, or prior GIB. He had no personal or family history of bleeding disorders.

On admission, vital signs were stable: blood pressure, 140/80 mm Hg; heart rate, 86/min; respiratory rate, 20/min; temperature, 98.6 °F; and oxygen saturation, 98% on room air. He appeared pale but was alert and oriented. Cardiovascular examination revealed an ejection systolic murmur best heard at the right second intercostal space radiating to the carotids and a soft second heart sound. Abdominal examination was unremarkable.

**Figure 1:**
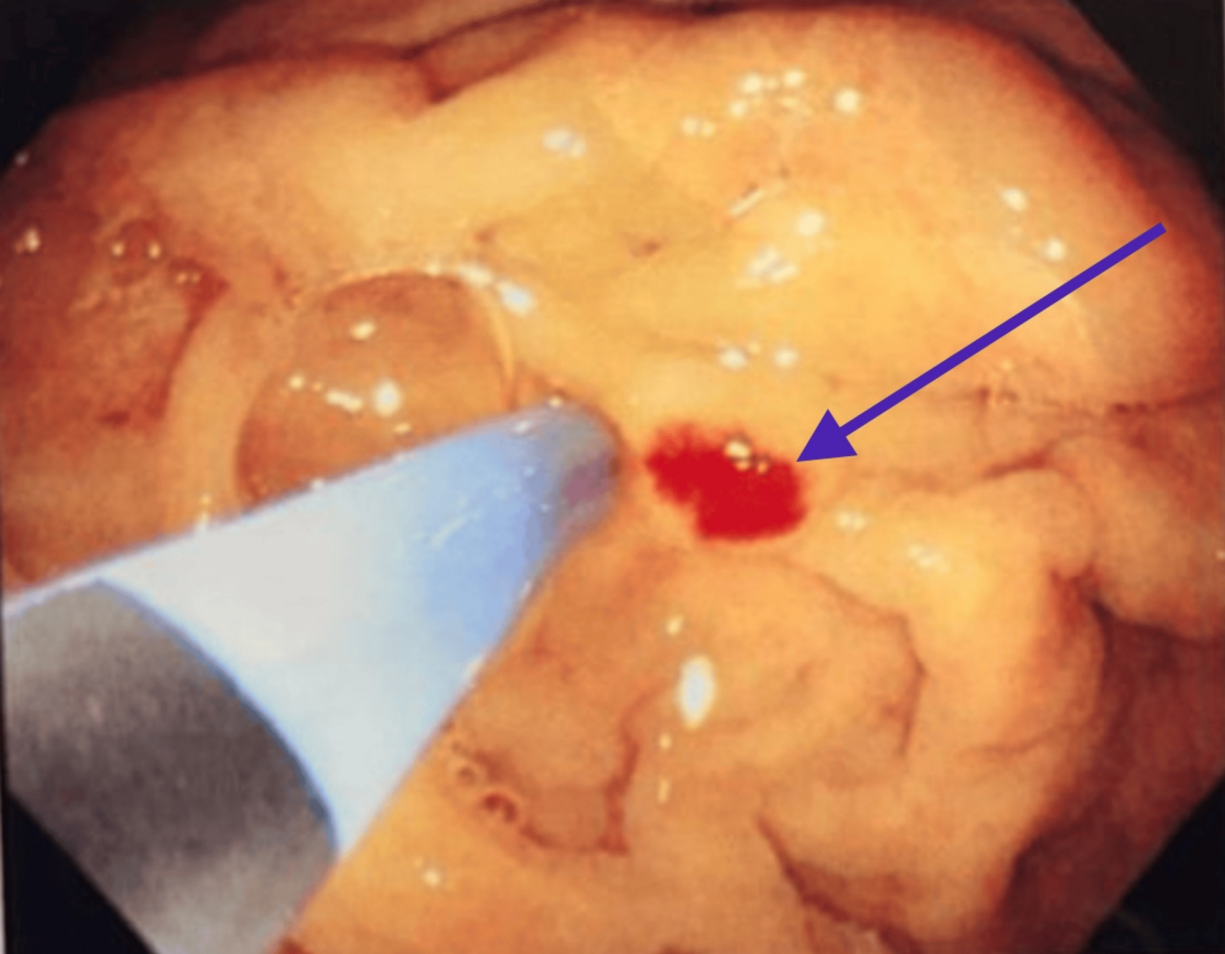
Endoscopic view showing angioectatic lesion in the stomach prior to intervention.

**Figure 2:**
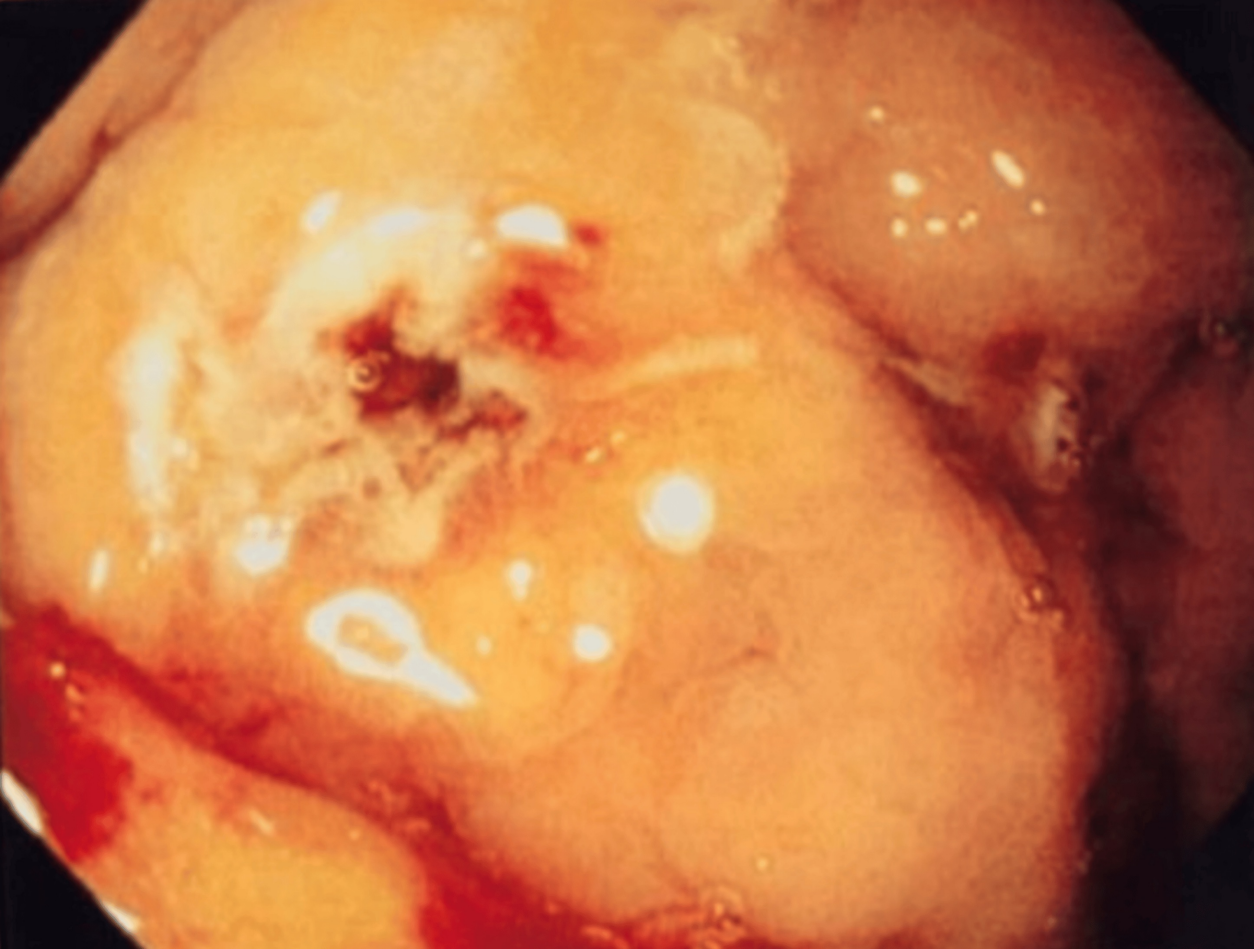
The same lesion following application of argon plasma coagulation, showing cauterized and hemostatic appearance.

Laboratory investigations (Table 1) showed a hemoglobin level of 8.6 g/dL and hematocrit of 25.6%. White blood cell count was 7490/mm^3^, and serum creatinine was 1.44 mg/dL. The electrocardiogram was within normal limits. Upper gastrointestinal endoscopy showed Los Angeles grade A esophagitis, a hiatal hernia, and angioectasia in the stomach (Figures 1, 2). Argon plasma coagulation (APC) was applied for hemostasis. Colonoscopy was performed and did not reveal any bleeding lesions. A previous echocardiogram report had documented severe calcific AS and mild AR. The aortic valve area was 0.6 cm², and the aortic jet velocity was 5.2 m/s.

**Table 1:** Laboratory investigations. WBC: white blood cell, RBC: red blood cell, PCV: packed cell volume, MCV: mean corpuscular volume, MCH: mean corpuscular hemoglobin, MCHC: mean corpuscular hemoglobin concentration, RDW-CV: red cell distribution width–coefficient of variation, MPV: mean platelet volume.

Test Name	Value	Unit	Reference Range
Haemoglobin	8.6	gm/dL	13.0 to 17.0
Total WBC count	7490	cells/mm^3^	4000 to 11,000
Neutrophils	67	%	40 to 70
Lymphocytes	24.3	%	20 to 40
Eosinophils	3.7	%	1 to 6
Monocytes	4.7	%	2 to 10
Basophils	0.3	%	0 to 1
Absolute neutrophils	5020	cells/mm^3^	1800 to 7700
Absolute lymphocytes	1820	cells/mm^3^	1000 to 4800
Absolute eosinophils	280	cells/mm^3^	20 to 500
Absolute monocytes	350	cells/mm^3^	200 to 1000
Absolute basophils	20	cells/mm^3^	0 to 200
RBC count	3.22	million/mm^3^	4.5 to 5.9
PCV/hematocrit	25.6	%	40 to 54
MCV	79.5	fL	80 to 96
MCH	26.7	pg	27 to 33
MCHC	33.6	g/dL	33 to 36
RDW-CV	14.5	%	11.5 to 14.5
Platelet count	234,000	/uL	150,000 to 450,000
MPV	9.2	fL	7.4 to 10.4
Sodium	139	mmol/L	135 to 145
Potassium	4.87	mmol/L	3.5 to 5.1
Urea	24	mg/dL	7 to 20
Creatinine	1.44	mg/dL	0.6 to 1.2

These findings were consistent with GAVE in the context of severe AS, suggestive of Heyde’s syndrome. Confirmation of acquired von Willebrand syndrome (AVWS) with ristocetin cofactor assay or vWF multimer analysis was not done due to the patient’s financial constraints.

After endoscopic treatment of the bleeding lesion, cardiology consultation was obtained, and he was referred for aortic valve replacement (AVR). He was discharged in stable condition with plans for follow-up and definitive surgical intervention (AVR). The patient was initially reluctant about surgical correction due to financial constraints but is currently following up with the cardiologist.

## Discussion

Heyde’s syndrome is a multifactorial clinical entity linking valvular heart disease, specifically AS, to GIB due to mucosal angiodysplasia, with the pathophysiological underpinning being AVWS [2]. High shear forces generated across the stenotic aortic valve result in the proteolytic cleavage of high-molecular-weight vWF multimers, impairing platelet adhesion and predisposing to mucosal bleeding [3].

The most common cause of bleeding in Heyde’s syndrome is angiodysplasia, with the most common source being the small intestine, followed by the cecum and ascending colon. The stomach as a bleeding source is rare in Heyde’s syndrome, as noted by Saha et al. in a systematic review, with 7.8% of the total sources being the stomach [4].

GAVE, initially defined by Rider et al. in 1953, refers to dilated blood vessels in the antrum that radiate to the pylorus [6,7]. It is also referred to as “watermelon stomach” because of its characteristic pattern comparable to the stripes observed on watermelons [5,7]. This condition is an unusual cause of upper GIB, accounting for about 4% of all non-variceal GI hemorrhages and 6% of upper GI hemorrhages in cirrhotic patients [3,7]. It was also noted in a retrospective study of 85,000 patients with GAVE that 6% of the cases had secondary AS, with a two-fold increase in risk for patients with AS to develop GAVE [8].

Diagnosis of Heyde’s syndrome includes identifying the typical clinical manifestations of severe AS, including syncope, angina, heart failure, systolic ejection murmur, and pulsus parvus et tardus. Echocardiography will confirm the clinical findings. Heyde’s syndrome demonstrates a selective deficiency of high-molecular-weight vWF. The laboratory diagnosis of vWD involves a platelet function assay (PFA) as an initial test that measures vWF antigen levels and vWF ristocetin cofactor activity. vWF multimer analysis is used as a confirmatory test if PFA is abnormal [9]. Testing was not performed in this case because of the patient’s financial constraints. This aligns with findings from a systematic review by Saha et al., which reported that AVWS was not documented in 50 of 77 Heyde’s syndrome cases, and vWF levels were either normal or not assessed in the majority (65%) [4]. This suggests that AVWS testing is a supportive tool but is not required for diagnosis. Although vWF studies are not mandatory for diagnosis, they can be useful in some situations, such as before and after aortic valve interventions to track the resolution of AVWS, and in persistent or recurrent bleeding even after valve intervention to reassess vWF multimer recovery and rule out residual AS or other bleeding causes [10]. GI angiodysplasia can be directly visualized using upper endoscopy or colonoscopy and treated with APC [7].

Definitive correction of the aortic valve disease with AVR, either surgical (SAVR) or transcatheter (TAVR), is associated with rapid recovery of the bleeding diathesis in Heyde’s syndrome and GIB cessation. By correcting the mechanical shear stress, AVR allows for the restoration of normal vWF multimer distribution, leading to hemostatic recovery. A 2023 meta-analysis showed an 87% recovery rate of AVWS and a 73% cessation rate of GI bleeding. vWF multimer profiles begin normalizing within 30 minutes post-intervention and show significant improvement within 24 hours [10].

Differences in efficacy exist between SAVR and TAVR in cessation of GI bleeding, with 82% of SAVR patients and 64% of TAVR patients achieving cessation of GI bleeding. However, no differences were reported in all-cause mortality and hospital charges in patients with Heyde’s syndrome who underwent either SAVR or TAVR. The TAVR cohort showed lower rates of stroke and blood transfusions, as well as more routine discharges [11]. Hence, one cannot make a strong recommendation for SAVR versus TAVR in Heyde’s syndrome based on the currently available literature, and the decision must be made on a case-by-case basis.

## Conclusions

Heyde’s syndrome remains an underdiagnosed yet treatable condition, typically characterized by the triad of AS, GIB, and AVWS. While intestinal angiodysplasia is the most frequently reported bleeding source, this case highlights GAVE as a rare but clinically significant manifestation. Diagnosis of Heyde’s syndrome is often delayed, sometimes by years, due to low clinical suspicion and the absence of standardized guidelines. As our case demonstrates, timely recognition of the syndrome, prompt endoscopic intervention, and planning for definitive AVR are essential components of effective management.

This case lends support to the hypothesis that GAVE may be an underrecognized variant of Heyde’s syndrome. Furthermore, most existing data on the efficacy of AVR in Heyde’s syndrome pertain to cases involving colonic angiodysplasia. There is a pressing need for future studies to explore the role and outcomes of AVR in patients with GAVE-associated Heyde’s syndrome. Coordinated multidisciplinary care involving cardiology and gastroenterology is crucial to optimizing outcomes in these presentations.
